# Effects of Soil Amendment With Wood Ash on Transpiration, Growth, and Metal Uptake in Two Contrasting Maize (*Zea mays* L.) Hybrids to Drought Tolerance

**DOI:** 10.3389/fpls.2021.661909

**Published:** 2021-05-20

**Authors:** Leila Romdhane, Leonard Barnabas Ebinezer, Anna Panozzo, Giuseppe Barion, Cristian Dal Cortivo, Leila Radhouane, Teofilo Vamerali

**Affiliations:** ^1^Laboratoire Sciences et Techniques Agronomiques (LR16INRAT05), National Institute of Agricultural Research (INRAT), University of Carthage, Ariana, Tunisia; ^2^Department of Agronomy, Food, Natural Resources, Animals and the Environment, University of Padua, Legnaro, Italy

**Keywords:** corn, drought tolerance, leaf transpiration, nutrient availability and uptake, phenolic acids, progressive water stress, root growth, wood ash

## Abstract

Wood ash as a soil amendment has gained wide spread acceptance in the recent years as a sustainable alternative to chemical fertilizers, although information regarding the effects of its application on maize growth and yield in the context of climate change and increasing drought severity is lacking till date. In the present study, field and pot trials were carried out at the experimental farm of the University of Padova at Legnaro (NE Italy) in a silty-loam soil in order to investigate the effects of soil amendment with wood ash (0.1% w/w, incorporated into the 0.2-m top soil) on the bioavailability of mineral elements and their uptake by maize. Characteristics analyzed included plant growth, leaf transpiration dynamics, and productivity in two contrasting hybrids, P1921 (drought sensitive) and D24 (drought tolerant). Wood ash contained relevant amounts of Ca, K, Mg, P, and S, and hazardous levels of Zn (732 mg kg^−1^), Pb (527 mg kg^−1^), and Cu (129 mg kg^−1^), although no significant changes in total soil element concentration, pH, and electrical conductivity were detected in open field. Ash application led to a general increasing trend of diethylene triamine penta-acetic acid (DTPA)-extractable of various elements, bringing to higher grain P in D24 hybrid, and Zn and Ni reductions in P1921 hybrid. Here, the results demonstrated that ash amendment enhanced shoot growth and the number of leaves, causing a reduction of harvest index, without affecting grain yield in both hybrids. The most relevant result was a retarded inhibition of leaf transpiration under artificial progressive water stress, particularly in the drought-tolerant D24 hybrid that could be sustained by root growth improvements in the field across the whole 0–1.5 m soil profile in D24, and in the amended top soil in P1921. It is concluded that woody ash can be profitably exploited in maize fertilization for enhancing shoot and root growth and drought tolerance, thanks to morphological and physiological improvements, although major benefits are expected to be achieved in drought tolerant hybrids. Attention should be payed when using ash derived by metal contaminated wood stocks to avoid any health risk in food uses.

## Introduction

Wood ash is the product of complete combustion of wood materials that can be resourced for plant nutrition and nutrient cycling in agriculture (Bhattacharya and Chattopadhyay, 2002). Containing adequate proportions of a variety of macronutrients and micronutrients, and a mixture of oxides, hydroxides, carbonates, and silicates (Eriksson, 1998; Demeyer et al., 2001), wood ash is potentially an excellent soil fertilizer that is gaining wide acceptance as an alternative of chemical fertilizers. Contributing to the circular economy, wood combustion, and valorization of the resultant wood ash as a soil amendment, is one of the most sustainable options available to meet the increasing demand for bioenergy and soil fertility amelioration in recent years (Huotari et al., 2015). Due to its liming effect, wood ash can also substitute liming products in counteracting soil acidification (Knapp and Insam, 2011; Arshad et al., 2012; Cruz-Paredes et al., 2017; Dvořák et al., 2017).

There are some negative effects reported after wood ash application, which can be attributed to its high alkalinity, low nitrogen content (Bramryd and Fransman, 1995), and possible presence of high amounts of toxic substance and heavy metals, especially Cd, depending on the quality of the initial wood stock (Levula et al., 2000; Demeyer et al., 2001; Perkiömäki et al., 2003; Pitmann, 2006). Hence, morphological and physiological damage to crops are imminent if over-applied. Nonetheless, when applied at appropriate levels, wood ash application has been reported to increase soil fertility by enhancing respiration and other metabolic activities of the soil microbial community (Fritze et al., 1994; Martikainen et al., 1994), and significantly improve growth and yield of some crops, especially maize (Roger and Sharland, 1997; Owolabi et al., 2003; Nottidge et al., 2005; Mbah et al., 2010). While these positive effects observed with wood ash application are due to its liming effect and higher availability of essential nutrients for plant growth promotion, increased water retention (Pathan et al., 2003; Stoof et al., 2010), and improved water infiltration (Yunusa et al., 2006) are some of its positive effects expected on soil hydraulic properties, although still little studied.

From climate change projections, it is apparent that most regions worldwide will be affected by more irregular rainfall distribution patterns and presumably longer dry periods with looming drought scenarios. Additionally, an increase in temperature, and soil degradation, will challenge agricultural production. Hence, sustainable intensive farming with more effective utilization of agricultural land and water resources will be required to meet the demands of a fast-growing population. Therefore, soil amendments including wood ash will be essential to drive sustainable agriculture practices by providing plant nutrition, improving soil hydrological characteristics, and promoting stress resilience in crops.

In addition to other complex morpho-anatomical, physiological, and biochemical changes, it is well known that drought stress in crops induces stomatal closure, which leads to reduced CO_2_/O_2_ ratio in leaves affecting photosynthesis. These conditions result in excess accumulation of reactive oxygen species (ROS) and, consequently, oxidative stress. As a countermeasure to mitigate oxidative stress, plants induce anti-oxidative enzymes as well as metabolites like glutathione, ascorbic acid, soluble sugars, and phenolic compounds (Efeoğlu et al., 2009; Nabeela et al., 2015). Previously, plants grown in wood ash amended soil showed an increased content of soluble sugars and accumulation of antioxidative enzymes, such as superoxide dismutase, ascorbate peroxidase, guaiacol peroxidase, and catalase (Chugh et al., 2011; Panda et al., 2020). In spite of the substantial evidence on wood ash to improve resilience to various abiotic stress, such as drought, the mechanism by which this occurs is not well understood. Although not thoroughly established, the improved stress resilience of plants grown in soil amended with wood ash may be linked to its ability to induce anti-oxidative mechanisms and it is still to be explored if such improved stress resilience is a result of a priming effect by the wood ash, along with the well-known improvements in nutritional and physico-chemical properties of the soil. While there are several studies on the effects of wood ash application in the forest ecosystem (Demeyer et al., 2001; Pitmann, 2006), there is still a lack of knowledge concerning crops, especially on the effects of wood ash application under abiotic stress condition.

Against this background, the present study innovatively investigated some morpho-physiological aspects of an essential staple cereal crop, such as maize, in a soil amended with wood ash in order to valorize this by-product. The specific objectives of this study were (i) to assess shoot and root growth and productivity of two maize hybrids (*Zea mays* L.) with different levels of drought tolerance, in response to the application of wood ash in open fields under rain-fed conditions; (ii) to evaluate the impact of ash amendment on the bioavailability and uptake of essential and non-essential elements by these two maize hybrids, and (iii) to study the leaf transpiration dynamics and root morphological parameters in both hybrids under progressive water stress in controlled pot conditions.

## Materials and Methods

### Materials and Plants Used

Wood ash used as soil amendment was produced from complete combustion of woody biomass of willow at 800°C constant temperature. To investigate the effects of ash as soil amendment, two commercial maize (*Zea mays* L.) hybrids with different responses to drought stress, i.e., P1921 (sensitive) and D24 (tolerant; Pioneer Hi-Bred, DuPont Co., United States; FAO class 700), were compared in field and pot experiments with a silty-loam soil containing 19% clay, 65% silt, 16% sand, 1.65% organic matter, 0.1% total N content, CEC of 11.4 cmol (+) kg^−1^, and pH 7.75.

### Field Experiment

The two hybrids (P1921 and D24) were cultivated for 156 days from May to October 2018 in a randomized block experimental design with two treatments (ash amended soil vs. untreated controls; *n* = 3) at the University of Padua’s Experimental Farm at Legnaro (NE Italy; 45° 21' 0.5'' N, 11° 56' 53.2'' E, 8 m a.s.l.). The site has a silty-loam soil (fulvi-calcaric-cambisol; USDA classification), with 1.7% organic matter, CEC of 11.4 cmol (+) kg^−1^, and total N content of 1.1 g kg^−1^ (arable layer). Wood ash at 0.1% w/w (i.e., 0.26 kg ash m^−2^) was mixed with the 20-cm topsoil (Ash), while untreated soil (Unt) served as control. Local suggested farming practices for maize were followed, and recommended doses of fertilizers (30 kg ha^−1^of N and 100 kg ha^−1^each of P_2_O_5_ and K_2_O) were added to the soil before sowing. Seeds were sown at 18 cm intervals along rows 75 cm apart (plot size 3 × 3.5 m, four rows per plot). The plots were dressed with a second dose of 170 kg N ha^−1^ in mid-June.

Plants were grown under natural rainfall without irrigation throughout the course of the experiment. During 2018, the climatic data (monthly rainfall and mean temperature) revealed warmer temperatures and good rainfall, with moderate water stress, particularly in June, compared with the historical 20-year mean. Soil samples (0–20 cm depth) were collected for each treatment (*n* = 3) at 10 days after sowing (DAS), and the soil samples were oven-dried at 105°C for 36 h and passed through a 2-mm sieve for successive chemical analysis (see below).

### Assessment of Plant Transpiration in Pot Experiment

A pot trial was set up specifically for assessing the effects of wood ash amendment on plant transpiration dynamics over the fraction of transpirable soil water (FTSW). The use of pots allowed to counteract the spatial variability of field conditions (e.g., water table depth and soil water holding capacity). The trial was arranged following a randomized block design with three replicates (*n* = 3). For each pot, wood ash at 0.1% (60 g ash) and 2.5 g of ternary 15-15-15 (NPK) fertilizer were mixed with 5 kg of soil. Tapered cylindrical pots (20 cm high × 20 cm top diameter × 15.3 cm bottom diameter, volume 5 L) were filled with ash-amended and untreated soil (Unt).

Three seeds of each maize hybrid (P1921 and D24) were sown in each pot on 11 August 2018 and after 1 week from germination; plants were thinned to one per pot. Thereafter, adequate soil moisture was maintained by periodical irrigation every 2–3 days with distilled water. At the fourth-leaf stage, the field capacity (32% w/w) was reached by abundant watering and percolation for 24 h. Adequate care was taken to prevent soil evaporation and water loss, by covering the pots with disposable plastic dishes, and sealing the bottom hole. In this way, it was ensured that the only way the plant could lose water was through transpiration, avoiding soil evaporation and percolation. Two holes were made in the plastic dishes used as cover, one around the plant stem (adequately sealed) and the other for irrigation.

Pots were then placed inside a greenhouse equipped with a temperature guided automatic roof that closed and opened when the temperature was <20 and >24°C, respectively. After the pots had been covered with plastic dishes, they were weighed daily, the first weight serving as the reference. Two water regimes were tested: (i) controls, which were irrigated daily to restore 100% of the transpired water, and (ii) progressive water stress, where the pots were not irrigated at all (drought conditions) until reaching the wilting point.

Daily transpiration was calculated as the difference between the pot weight on each day of the experiment (at approximately 9 a.m.) and on the previous day. Relative Transpiration (RT) was calculated as the ratio between daily transpiration and estimated daily potential transpiration. For greater accuracy, transpiration of full irrigated controls was not used directly as potential transpiration, but was estimated as proposed by Vamerali et al. (2003). Plant growth was negligible compared to the overall amount of water transpired during the experiment. The daily FTSW was estimated with Eq. (1):

$$\mathrm{Daily FTSW}\;\left(\%\right)=\frac{\mathrm{Daily}\;\mathrm{pot}\;\mathrm{weight}-\mathrm{Final}\;\mathrm{pot}\;\mathrm{weight}}{\mathrm{Initial}\;\mathrm{pot}\;\mathrm{weight}-\mathrm{Final}\;\mathrm{pot}\;\mathrm{weight}}\;\times \;100$$ (1)

The RT was plotted against the FTSW for 29 days and fitted with a plateau linear regression model expressed by the following Eq. (2):

$$Y=a+b\times \left(x-c\right)\times \left(x\leq c\right)$$ (2)

where *Y* is the relative transpiration (RT, %), *x* is the daily FTSW, *a*, *b*, and *c* are empirical coefficients, and *a* representing the maximum RT (plateau) and *c* the FTSW threshold at the beginning of RT decline.

### Chemical Analyses

Extracts of a 1:2 w/v soil-to-water mixture (10 g of dried soil in 20 ml distilled water) after shaking for 2 h and filtering through Whatman No. 42 filter paper were used to measure soil electrical conductivity with an EC-pH meter (HI255 Combined Meter, Hanna Instruments, Rhode Island, United States). Soil pH was measured in 1:2.5 w/v soil-to-water suspensions (10 g of soil in 25 ml distilled water).

Following the EPA 3051 method (USEPA, 1995a), total element concentrations in the wood ash and the soils (Unt and Ash) were determined in microwave (Mileston ETHOS 900, Bergamo, Italy) acid-digested samples (~0.4 g of soil, *n* = 3) after dilution to 25 ml with distilled water and filtering with 0.45 μm cellulose-acetate membrane filters. Diethylene triamine penta-acetic acid (DTPA)-extractable elements were detected in 50-g samples of homogenized dried soil (*n* = 3) after shaking for 2 h (60 cycles min^−1^) with a 100-ml solution (pH 7.3) of DTPA (1.97 g L^−1^), calcium chloride bihydrate (1.46 g L^−1^), and triethanolamine (14.92 g L^−1^), and finally centrifuged at 2,599 × *g* for 5 min. Following the same procedures as for DTPA extraction, water-soluble elements extracted using Milli-Q water were also detected in wood ash samples prior to soil amendment (*n* = 3).

Total and DTPA/water-extractable P, Mg, Ca, K, S, Cu, Zn, Co, Ni, Pb, and Cd were measured by ICP-OES using a certified reference material for each element (ERM-CC141, JRC-IRMM, Belgium).

### Plant Analyses

In the field trial, using a Minolta SPAD-502 chlorophyll meter (Konica-Minolta, Inc., Co., Hong Kong) and following the method described by Richardson et al. (2002), an index of chlorophyll content of the last fully-developed leaf (mean of three plants per plot, five sub-measurements per leaf) was measured once a week from mid-June to mid-September (leaf senescence). Plant height and number of leaves, and shoot and root biomasses (*n* = 3, one plant per plot) were recorded at silking (end of July), at the same time of root investigations.

For assessing yield components and nutrient composition, at harvest, one plant per plot was collected, divided into stem, leaves, cob, and grains, oven-dried at 105°C for 24 h, and thereafter weighed. According to the EPA 3052 method (USEPA, 1995b), approximately 0.4 g DW of milled sample plant material was microwave acid-digested using 7 ml HNO_3_ (65% v/v) and 1 ml H_2_O_2_ (30% v/v). Following mineralization, the samples were diluted to 25 ml with distilled water and filtered through 0.45 μm cellulose-acetate membrane syringe filters. The nutrient composition was analyzed by ICP-OES using a certified reference material for each element to ensure measurement accuracy.

Grain yield was assessed in the two central rows of each plot by manual harvesting. Near-infrared reflectance spectroscopy (NIRS, Infratec 1241 Grain Analyzer, Foss Analytical AB, Sweden) was used to determine the protein, lipid, and starch contents of the grains (*n* = 3; one per plot).

### Root Investigations

To investigate the root systems at the silking stage, one soil core (70 mm diameter, 1.5 m deep) was sampled from the inter-row 0.1 m from the stem of one maize plant per plot. Each soil core was split into 0.1 m-deep subsamples to describe the rooting profile. The roots were separated from the soil by flotation with a hydraulic centrifugation device and collected in a 500-μm mesh sieve, and then stored at 4°C in ethanol solution (12% v/v) before analysis (do Rosário et al., 2000). A flatbed scanner (EPSON Expression 11000XL, Canada) was used to acquire TIFF-format images of the roots at 1-bit 400 DPI resolution, and WinRHIZO ver. 2007b image analysis software (Regent Instruments Inc., Canada) with a threshold area > 30 pixels (0.0015 cm^2^) for analyzing root length, projected area, and diameter. Root length and area were measured per unit of ground surface for each depth interval to obtain the root length index (RLI, cm cm^−2^) and root area index (RAI, cm^2^ cm^−2^), respectively. For each depth interval, root length was also converted into volumetric root length density (RLD, cm cm^−3^).

Roots from subsamples were pooled prior to oven-drying (at 105°C for 36 h) and weighting for assessing root biomass.

The dynamics of root growth were also monitored during the growth cycle by the non-destructive electrical capacitance method. Data were acquired weekly by a handheld digital electrical capacitance meter (ELC-131D, Escort Instruments Corporation, Taipei, Taiwan) in a circuit composed of a negatively-charged electrode (applied at the stem base), roots, soil solution, and a positively-charged electrode (earthed in the soil near the plant; Dalton, 1995). The instrument settings were (i) positively-charged electrode: vertical stainless steel rod, 10 mm diameter, 550 mm long, 400 mm inserted into the soil 50 mm away from the stem base of one maize plant, and cable connection 150 mm above-ground level; (ii) negatively-charged electrode: fine stainless steel needle, orthogonally inserted into the stem base at a height of 60 mm (1-mm penetration into plant tissues). Capacitance values (pF) were recorded at 1 kHz frequency, with three replicates.

### Assessment of Free Phenolic Acids

Maize grains were milled to a fine powder, mixed thoroughly by sieving through a 60-μm mesh size screen, and stored at −20°C until analysis. Moisture content was determined by the difference in weight of the samples before and after oven-drying at 105°C for 36 h.

Free phenolic acids, such as *p*-coumaric acid, caffeic acid, syringic acid, vanillic acid, and *t*-ferulic acid were extracted according to the modified procedure by Adom and Liu (2002) and Adom et al. (2003). Specifically, a 0.1 g sample was extracted with 5 ml of 80% v/v chilled acetonitrile (ACN) in 10 ml tubes. The mixture was shaken for 5 min at 70 rpm at room temperature, and then centrifuged for 10 min at 10,000 × *g*. The supernatant was filtered at 0.2 μm (Acrodisc syringe filters with GHP membranes) and kept in clean tubes at −20°C until processing. The analysis was performed by High-Performance Liquid Chromatography (HPLC, Shimadzu Corporation, Kyoto, Japan) using an Ultra Tech sphere C18 column (1.5 μm, 33 mm × 4.6 mm; CIL Cluzeau, Sainte-Foy-La-Grande, France) at 36°C, phenolic acids being identified with a Shimadzu SPD-M20A Photodiode Array Detector at 282 nm wavelength. The mobile phase was 0.25% (v/v) trifluoroacetic acid (TFA, solvent A) and pure ACN (solvent B) delivered at a flow rate of 1.1 ml min^−1^ using a Waters 600E quaternary pump following a specific linear gradient programme (Dal Cortivo et al., 2017). Two-microliter samples were injected using a SIL 20 AC Prominence autosampler. The identification peak was confirmed on the basis of the retention time and absorbance spectrum of pure compounds (*p*-coumaric acid, caffeic acid, syringic acid, vanillic acid, and *t*-ferulic acid). Phenolic acid concentrations were calculated using standard calibration curves with determination coefficients >99% and expressed in units of μg g^−1^ on a DW basis.

### Experimental Design and Statistical Analysis

All the pot and field experiments were carried out in a randomized block experimental design with two treatments (ash amended soil vs. untreated controls). Normality and variance homogeneity of the data was tested and statistical differences among the treatment means were determined using an ANOVA and the Newman-Keuls test (*p* ≤ 0.05). Statistical analysis of all data was performed using the Statgraphics Centurion XVII software (Manugistic, Rockville, MD, United States).

## Results

### Physicochemical Properties of Wood Ash and Soil

Physicochemical characterization of ash used in field experiment showed an alkaline reaction (pH = 12) and high electrical conductivity (17 dS m^−1^). The wood ash had important amounts of macro‐ and micro-nutrients, such as K, P, S, Ca, Mg, and Cu. Two elements were present in excess compared to Italian Guideline Values (IGV) for agriculture amendments (Legislative Decree n.75/2010): the micronutrient Zn by 46.5% and the toxic metal Pb by 276.5% (Table 1). In ash, the bioavailability was high for K (51% of total in both water and DTPA-extraction solution) and S (62% in DTPA and 64% in water-extraction solution), while it was low in Cu (3% in DTPA-extraction), and negligible (<1%) for the other elements.

**Table 1:** Total element concentrations in ash and their availability after extraction in water and in diethylene triamine penta-acetic acid (DTPA)-trietanolammine-CaCl_2_ solution (mean ± SE; *n* = 3).

Element	IGV^1^ (mg kg^−1^)	Total (mg kg^−1^)	DTPA-extractable		Water-extractable	
			mg kg^−1^	%^2^	mg kg^−1^	%^2^
P		19,566 ± 150	0.18 ± 0.04	0.00092	0.19 ± 0.01	0.00097
K		56,650 ± 830	29,210 ± 873	51.56	28,960 ± 660	51.12
Ca		297,240 ± 5,000	-	-	70 ± 5	0.024
Mg		31,930 ± 730	-	-	-	-
S		6,210 ± 270	3,860 ± 140	62.16	3,950 ± 140	63.61
Zn	500	732 ± 4.15	6.59 ± 0.300	0.9	0.79 ± 0.007	0.1
Cu	230	129 ± 2.784	3.84 ± 0.077	2.98	0.17 ± 0.01	0.13
Ni	100	5.28 ± 0.38	0.038 ± 0.006	0.71	0.012 ± 0.002	0.22
Co		3.37 ± 0.13	0.031 ± 0.0007	0.92	-	-
Cd	1.5	1.07 ± 0.09	0.007 ± 0.0005	0.65	-	-
Pb	140	527 ± 74.98	0.09 ± 0.00268	0.01	0.82 ± 0.08	0.15

1 IGV: Italian Guideline Values for agriculture amendments according to Italian Legislative Decree n.75/2010.

2 Percentage on total concentration.

In the field experiment, the applied rate of ash did not alter the concentrations of total elements and bioavailable fractions significantly, although increases in total concentrations of P, K, Zn, and Cu (<10%) and bioavailable fractions of K, Mg, and Cd (18, 13, and 3%, respectively) were observed compared to untreated controls. Soil pH and electrical conductivity did not show significant alterations after ash addition (Table 2).

**Table 2:** Field trial: total and DTPA-extractable element concentration, pH, and electrical conductivity in ash-amended soil (Ash, 0.1% w/w) vs. reference untreated soil (Unt; mean ± SE; *n* = 3).

Parameter	Unt			Ash		
	Total	DTPA-extractable	%^1^	Total	DTPA-extractable	%^1^
P mg kg^−1^	603 ± 4.74^a^	0.73 ± 0.08	0.12	620 ± 11.80^a^	-	
K mg kg^−1^	4,510 ± 218^a^	8.5 ± 0.7^a^	0.19	4,950 ± 510^a^	10 ± 0.7^a^	0.2
Ca mg kg^−1^	65,800 ± 3487^a^	-		64,300 ± 4020^a^	-	
Mg mg kg^−1^	31,600 ± 1280^a^	53 ± 1^b^	0.16	30,920 ± 1260^a^	60 ± 1^a^	0.19
S mg kg^−1^	184 ± 5.37^a^	20.30 ± 3.47^a^	11.03	181 ± 3.76^a^	22.31 ± 3.00^a^	12.32
Zn mg kg^−1^	65.9 ± 0.43^a^	0.98 ± 0.12^a^	1.47	68.3 ± 1.70^a^	1.23 ± 0.06^a^	1.80
Cu mg kg^−1^	21.8 ± 0.20^a^	3.55 ± 0.067^a^	16.23	22.3 ± 0.64^a^	3.72 ± 0.085^a^	16.68
Ni mg kg^−1^	17.0 ± 0.46^a^	0.40 ± 0.03^a^	2.35	17.2 ± 0.508^a^	0.50 ± 0.07^a^	2.90
Co mg kg^−1^	7.50 ± 0.08^a^	0.07 ± 0.001^a^	0.94	7.54 ± 0.164^a^	0.07 ± 0.001^a^	0.92
Cd mg kg^−1^	0.30 ± 0.02^a^	0.07 ± 0.0006^a^	23.4	0.27 ± 0.0063^a^	0.072 ± 0.001^a^	26.7
Pb mg kg^−1^	14.5 ± 74.98^a^	2.47 ± 0.11^a^	17.03	14.9 ± 0.544^a^	2.71 ± 0.09^a^	18.18
pH	7.75 ± 0.10^a^			7.65 ± 0.05^a^		
EC dS m^−1^	0.502 ± 0.026^a^			0.500 ± 0.022^a^		

1 Values are percentage of DTPA-extractable fraction on total concentration. Different letters in each row indicate significant differences between treatments within the same parameter (Newman-Keuls test, *p* ≤ 0.05).

### Plant Growth and Yield

In the field trial, significant increment in the leaf number was evidenced in both the maize hybrids D24 (+4.18%) and P1921 (+7.26%) after ash application (Table 3). Although the differences were not statistically significant, all the measured growth parameters, at least with D24, showed an increasing trend at flowering with ash application. Instead, in P1921, the main growth parameters, i.e., shoot weight, mean RLI, and RAI, showed an opposite trend, with slight reductions with ash amendment (Table 3). Interestingly, in both hybrids, the length fraction of finer roots increased with ash amendment.

**Table 3:** Field trial: growth parameter at flowering stage (mean ± SE; *n* = 3) in two maize hybrids (D24 and P1921) grown in ash-amended soil (Ash, 0.1% w/w) vs. untreated controld (Unt).

Hybrid	Treatment	Shoot biomass (g DW)	Number of leaves	Plant height (last node; cm)	Root biomass (mg DW cm^3^)	Mean RLI (cm cm^−2^)	Mean RAI (cm^2^ cm^−2^)	Mean root diameter (μm)	Root length with Ø ≤ 250 μm (% on total length)
D24	Unt	170 ± 14^a^	12.9 ± 0.20^b^	253 ± 2.20^a^	0.07 ± 0.01^a^	10.4 ± 0.75^a^	1.26 ± 0.06^a^	355 ± 15.0^a^	46.8 ± 3.66^a^
	Ash	194 ± 16.39^a^	13.44 ± 0.17^a^	253.33 ± 3.01^a^	0.08 ± 0.01^a^	12.1 ± 1.46^a^	1.48 ± 0.28^a^	405 ± 41.66^a^	49.33 ± 5.78^a^
P1921	Unt	176 ± 26^a^	11.7 ± 0.33^b^	246 ± 3.14^a^	0.09 ± 0.001^a^	14.2 ± 1.23^a^	1.72 ± 0.08^a^	386 ± 15.3^a^	44.7 ± 3.28^a^
	Ah	171 ± 16^a^	12.55 ± 0.17^a^	255.44 ± 4.21^a^	0.09 ± 0.003^a^	13.4 ± 0.81^a^	1.55 ± 0.07^a^	340 ± 14.9^a^	53.66 ± 2.02^a^

Different letters indicate statistically significant differences between treatments within the same hybrid (Newman-Keuls test, *p* ≤ 0.05). Root parameters across the 0–150 cm soil profile: RLI, root length index; RAI, root area index. Different letters indicate statistically significant differences between treatments (Newman-Keuls test, *p* ≤ 0.05).

**Figure 1:**
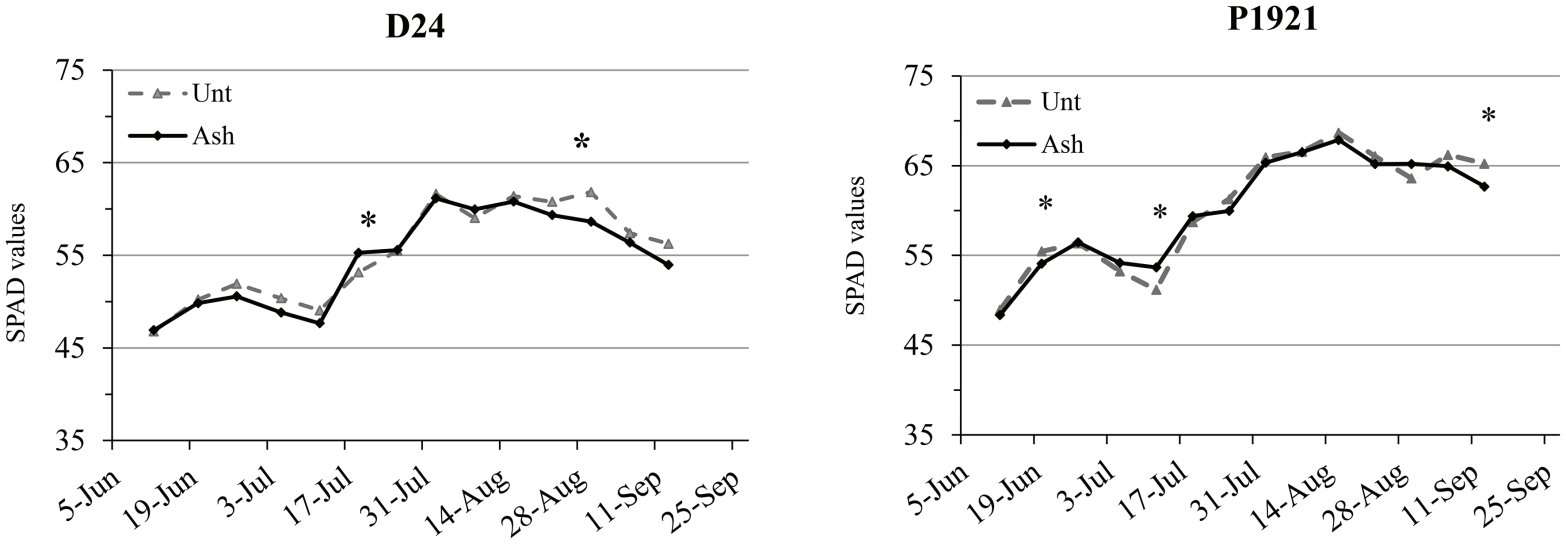
Field trial: dynamics of soil plant analysis development (SPAD) values as an index of leaf chlorophyll content in two maize hybrids (D24 and P1921) grown in ash-amended soil (Ash, 0.1% w/w) vs. untreated controld (Unt; *n* = 3). Asterisks indicate statistically significant differences between treatments within the same observation date (Newman-Keuls test, *p* ≤ 0.05).

Dynamics of leaf chlorophyll content, expressed as soil plant analysis development (SPAD) values, indicated that the ash application did not have any significant improvement during the vegetative phase in the hybrid D24, while led to a decrease at the end of August (Figure 1). In the hybrid P1921, the dynamics of SPAD values was inconsistent and showed a decrement in mid-June and an increase in mid-July (Figure 1).

**Figure 2:**
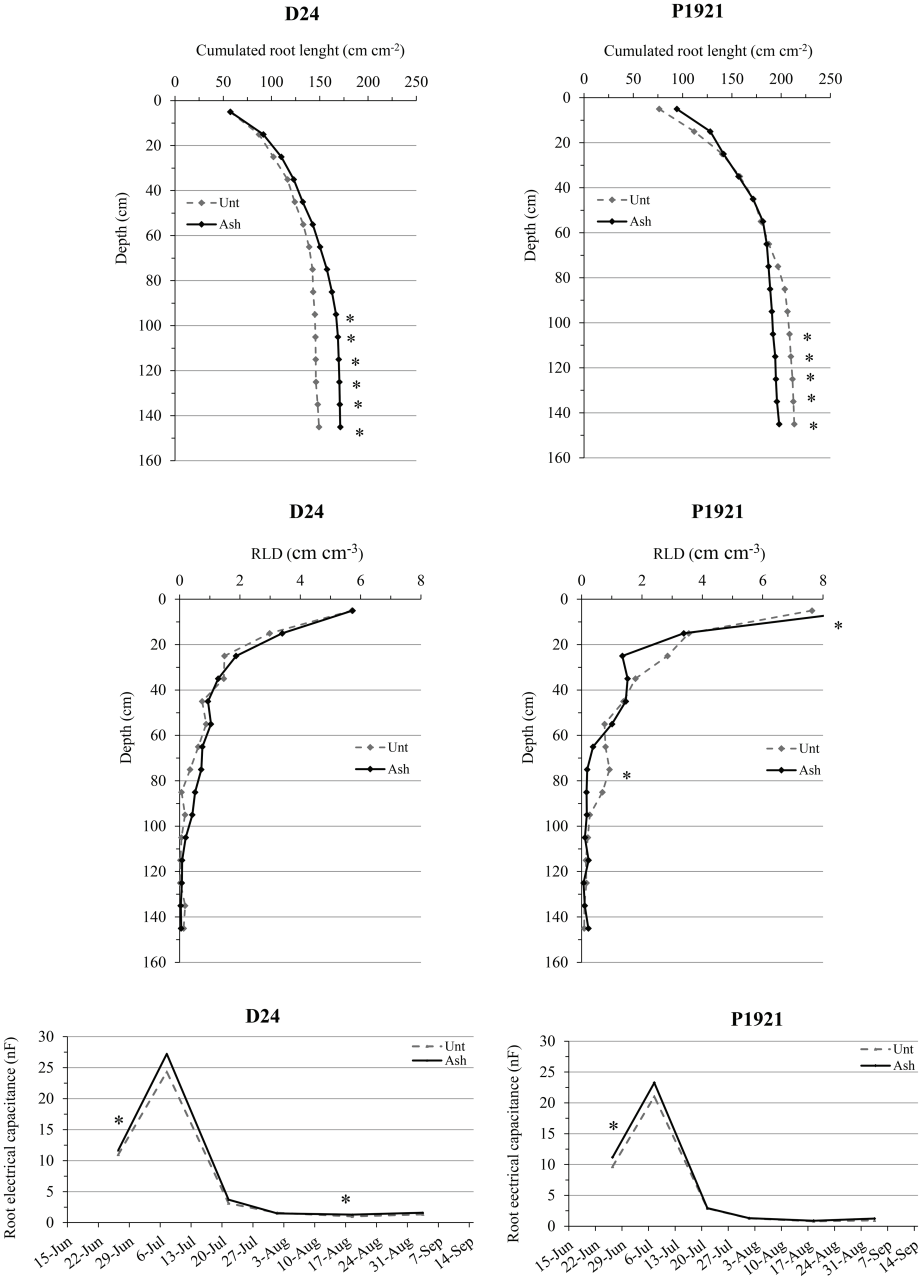
Field trial: cumulated root length, root length density (RLD) pattern, and root electrical capacitance in two maize hybrids (D24 and P1921) grown in ash-amended soil (Ash, 0.1% w/w) vs. untreated controls (Unt; *n* = 3). Asterisks indicate statistically significant differences between treatments (Newman-Keuls test, *p* ≤ 0.05).

Ash application improved the RLD of the hybrid P1921 at 10–20 cm depth (9.75 vs. 7.75 cm cm^−3^ in untreated soil) and at 70–100 cm (0.9 cm cm^−3^ compared to 0.1 cm cm^−3^ in untreated soil) layers (Figure 2). In D24, the rooting profile was not significantly influenced by ash application, although RLD was generally enhanced by ash, especially within 50–100 cm depth, resulting in greater cumulated root length across the whole soil profile (Figure 2). The root electrical capacitance, which is an indicator for the living active root of the plants, was measured starting from June. Both D24 and P1921 revealed early increases in root EC with ash amendment at end June (12-leaf stage; +16% on average), and at mid-August with the beginning of physiological maturity in the drought tolerant hybrid (D24; +23%; Figure 2).

**Figure 3:**
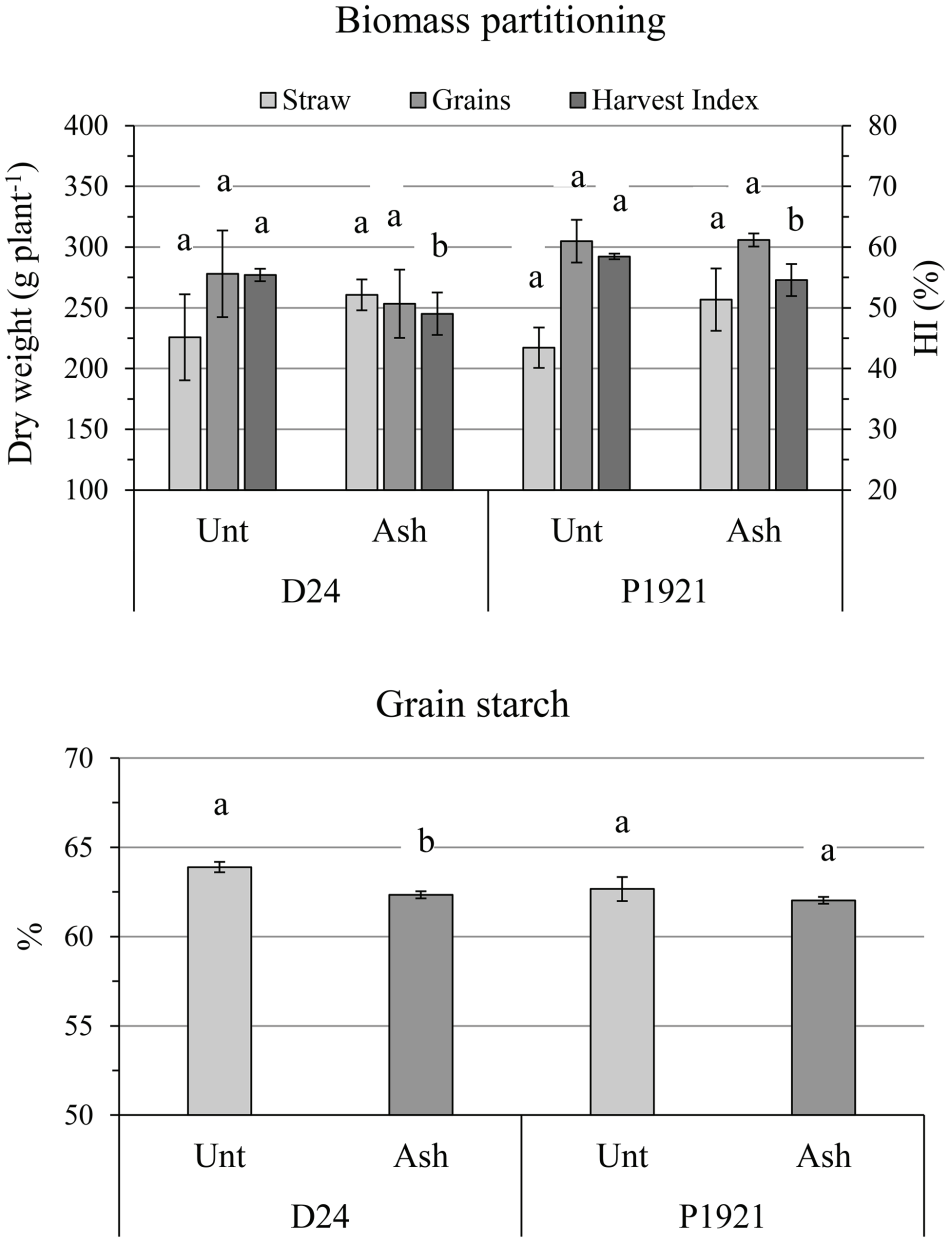
Field trial, harvest time: straw DW (stem + leaves + cobs), grain DW, harvest index (HI), and starch content in kernels of two maize hybrids (D24 and P1921) grown under ash-amended soil (Ash, 0.1% w/w) vs. untreated controls (Unt; mean ± SE, *n* = 3). Different letters indicate statistically significant differences between treatments within the same hybrid (Newman-Keuls test, *p* ≤ 0.05).

The addition of ash did not influence significantly yield and the dry weight of grains per plant, but the hybrid D24 showed a tendency of decrease (−5 and −9%, respectively; Figure 3; Supplementary Table SI1). The harvest index in both hybrids showed a significant decrement (−11.5% for D24 and −6.7% for P1921; Figure 3). Accordingly, the straw dry weight (cob + stem + leaves) tended to increase in both D24 (+15%) and P1921 (+19%), however, the differences were not statistically significant (Figure 3).

**Figure 4:**
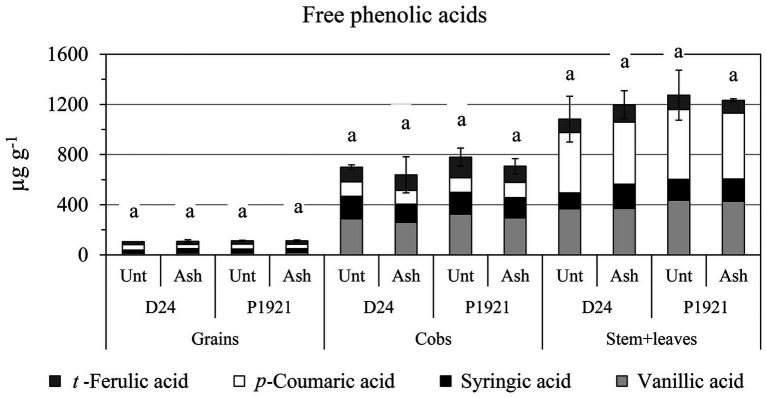
Field trial: concentrations of free phenolic acids in maize grains, cob, and straw (stem + leaves) of two maize hybrids (D24 and P1921) grown in ash-amended soil (Ash, 0.1% w/w) vs. untreated controls (Unt; mean ± SE for total concentration). Different letters within hybrids indicate statistically significant differences between treatments for total concentration (Newman Keuls test, *p* ≤ 0.05).

As regard kernel composition, a significant reduction in starch content was observed in D24 (−2%, absolute variation), which was also noticeable in P1921 (−1%) after ash application (Figure 3). Lipid and protein contents were not significantly influenced, although a general decreasing trend was observed with ash application (Supplementary Table SI1). Soil amendment had also a differential impact on the free phenolic acid content with respect to different plant parts of the two hybrids. An increase in trend was observed in the straw (stem + leaves) of the hybrid D24, especially for *p*-coumaric acid (Figure 4). Comparatively lower levels of free phenolic acids were noticed in cobs, followed by grains, wherein *p*-coumaric acid content was the most reduced after ash application among others.

### Element Content in Plants

**Figure 5:**
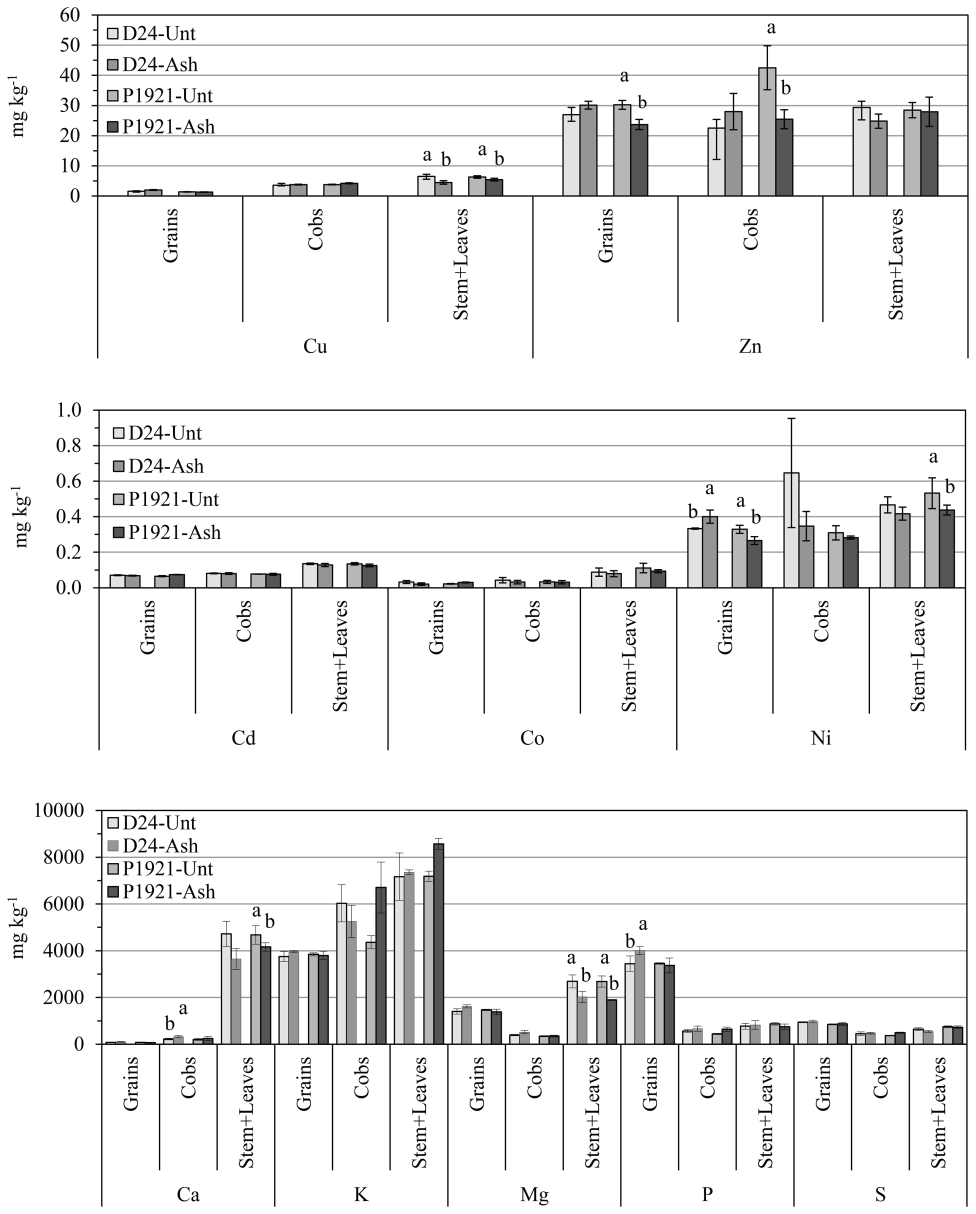
Field trial: element concentrations in grains, cob, and stem + leaves of two contrasting maize hybrids (D24 and P1921) grown in ash-amended soil (Ash, 0.1% w/w) vs. untreated controls (Unt). Differences between treatments (Newman Keuls test, *p* ≤ 0.05) within the same hybrid have been indicated with different letters (only when significant). Lead (Pb) was below the detection limit (b.d.l. < 0.003 mg kg^−1^ DW).

In the field trial, ash application had a significant impact on the plant uptake and accumulation of several elements, and differences were also evident among plant compartments. In the straw (stem + leaves), ash application resulted in significantly decreased concentrations of Cu (D24: −31%; P1921: −14%) and Mg (D24: −29%; P1921: −25%) in both the hybrids, according with increased growth and element dilution. Significant decrease in concentrations of Ca (−11%) and Ni (−19%) was also observed in P1921 (Figure 5). In cobs, increased concentration of Ca (+49%) in D24 and decreased Zn concentration in P1921 (−40%) were noticed. In grains, ash application resulted in increased concentration of P (+16%) and Ni (+21%) in D24, and Zn and Ni reductions (~21%) in P1921 (Figure 5).

### Leaf Transpiration Dynamics Under Progressive Water Stress

**Figure 6:**
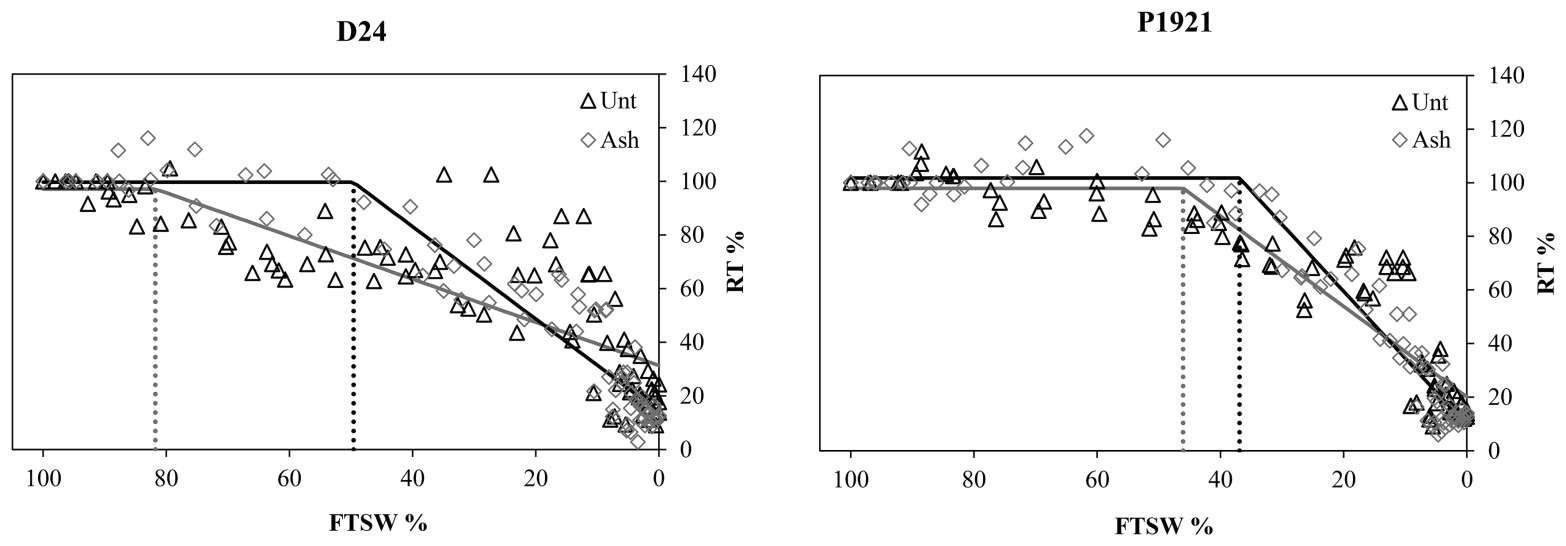
Pot trial: dynamics of relative transpiration (RT) over the fraction of transpirable soil water (FTSW) of maize hybrids (D24 and P1921) grown in ash-amended soil (Ash, 0.1% w/w) vs. untreated controls (Unt) under progressive water stress. Regressions with the Linear-Plateau model. Coefficient *c* (critical point) indicated by the dotted line.

In the pot trial, the effects of water stress were evaluated by calculating the “linear plateau regression” obtained by relating daily RT over the FTSW (Figure 6). Results showed that the linear plateau regression model of D24 grown in unamended soil starts to decline earlier (82% FTSW) than in P1921 (45% FTSW). Plants grown under ash addition transpired at their maximum down to lower FTSW, i.e., 50 and 38% in D24 and P1921, respectively, compared with their respective unamended controls (Figure 6).

## Discussion

### Soil Characteristics and Nutrient Availability

Plant growth and productivity is primarily dependent on the nutrient availability and physico-chemical properties of soil, which is dependent on soil EC and pH (Wolfe et al., 1990). Wood ash, a well-known soil corrective and fertilizer, is known from previous studies to improve the physicochemical properties and nutrient availability of the soil. Although transient increases of soil EC and pH were observed in previous experiments at double ash rate compared to ours (Lucchini et al., 2014), here it is evident that ash application did not adversely affect the soil EC in our experimental conditions (Table 2). The pH of the wood ash (pH = 12) used in the present study was comparable to previously reported studies (Demeyer et al., 2001; Kuokkanen et al., 2009; Nabeela et al., 2015). Albeit, the highly alkaline nature of the ash used as amendment in our trials, the soil pH was not significantly altered (Table 2). While wood ash is known for its liming effect by increasing the pH of acidic soils, several reports are available wherein there was no significant changes observed in the soil pH after ash application (Wheeler, 1997; Edmeades and Ridley, 2003; Arshad et al., 2012), while several others did at least temporarily (Adekayode and Olojugba, 2010; Lucchini et al., 2014). Changes in soil pH as a result of liming are dependent on various factors like the method of application, quantity and quality of the amendment, which includes the neutralizing value, particle size, and hardness affecting dissolution rates among others. It is also known that the liming effect can be dependent on the degree of precipitation and mineralogical make-up of the soil type. Hence, the expected change in soil pH after ash application could be counteracted by some of these factors resulting in no net change in pH as observed in our study and others previously reported.

The wood ash used in the present study had high Ca, K, Mg, P, Zn, and Cu and its composition was comparable to an extent with previous reports (Demeyer et al., 2001; Chirenje et al., 2006; Adekayode and Olojugba, 2010; Serafimova et al., 2011). Wood ash in our study had high concentration of macroelements in the order of Ca > K > Mg > P, as also generally reported (Demeyer et al., 2001). Among microelements, Zn had the highest concentration as compared to an inventory of 26 wood ash analyses from 15 different sources reported by Someshwar (1996). Bioavailability of important elements such as K and S was significantly high in our willow wood ash. Chemical composition and properties of wood ash can considerably vary and depend on several factors, including plant/tree species, plant part used for combustion, type of waste (wood, pulp, or paper residue), combustion conditions, collection and storage, soil type, and climate among several others (Campbell, 1990). As a consequence, properties and concentration of macro and microelements in wood ash can be very variable (Demeyer et al., 2001). Although toxic elements like Pb and Cd were present in ash, their bioavailability was negligible compared to the total concentration (Table 1). This was also observed in the ash amended soil of our field experiment (Table 2), suggesting that these toxic elements were immobilized in ash. Ash increased only the bioavailable fraction of Mg in the field experiment. High alkalinity of the wood ash used in the present study indicates that the majority of the macro‐ and micro-elements must be in the form of carbonates, hydroxides, and oxides (Nurmesniemi et al., 2008), complexed with cations, especially Ca. This can be correlated with the low bioavailability of most of the elements in wood ash (Table 1), which was also reflected in the field experiment (Table 2).

### Maize Growth, Yield, and Grain Quality

Ash amendment has often been reported to have an improvement of plant growth and yield in many crops, including maize (Erich and Ohno, 1992; Demeyer et al., 2001). In the present study, ash application increased significantly the leaf number in both the maize hybrids D24 and P1921, and an increasing trend was observed with most of the other growth parameters measured. Ash application had positive effects on RLD, total root length, and root electrical capacitance, however, some of the parameters had contrasting trends between the hybrids (Figure 2). With regard to dynamics of SPAD values, ash application did not have any significant improvement and instead showed a decrement in both the hybrids, particularly later in the season (Figure 1); neither was there any significant change in yield and the dry weight of grains per plant after ash application (Figure 3; Supplementary Table SI1). However, the straw dry weight (cob + stem + leaves) showed a tendency to increase in D24 (+15%) and P1921 (+19%) with decrement of harvest index. Taken together (whole plant), ash application did not significantly improve the maize growth and yield. Similar results have been reported earlier wherein either there was no effect on the growth (McDonald et al., 1994) or even a slight decrease in growth during the initial duration (Ferm et al., 1992). This could be attributed to the mineral composition of the ash, it having high concentrations of important elements but virtually no nitrogen. One of the main mechanisms for metals sorption by wood ash is the formation of precipitates (Chirenje et al., 2002, 2006; Voegelin and Kretzschmar, 2005; Cao et al., 2011). Recently, Ondrasek et al. (2021) showed that wood ash application shifted the rhizosphere biogeochemistry toward chemisorption/precipitation reactions influencing the soil-plant metal immobilization process. Hence, there is the possibility that the exogenous N provided by fertilizers and other important elements in the soil could be immobilized and trapped by reacting with the wood ash, as it usually has high soluble concentrations of carbonates and phosphates. Recent evidence also shows that wood ash application decreased the activity of dehydrogenase, acid phosphatase, and β-glucosidase, as well as fluorescein diacetate (FDA) activity, indicating reduced soil microbial activity (Pukalchik et al., 2018). Furthermore, wood ash application may strongly influence the soil texture, aeration, and water holding capacity, consequently having an impact on root growth dynamics leading to a range of possible effects on plant growth. However, there is mounting evidence for wood ash application to improve plant growth and yield (Demeyer et al., 2001; Mercl et al., 2016; Jagodzinski et al., 2018).

Regarding the grain quality, ash application reduced the starch content in both the hybrids and did not adversely affect the protein and lipid content compared to untreated control (Figure 3). Starch being the end product of photosynthesis and associated with carbon metabolism, reduced starch content could be correlated to the decrement observed in the SPAD values, which is proportional to the amount of chlorophyll present in the leaves and photosynthetic activity. This could also be attributed to reduced bioavailability of elements in soil, such as potassium, essential for starch metabolism through the activation of enzymatic systems, although not by our results.

Plant phenolics play pivotal roles in almost all plant growth and development processes with their antioxidant role. Phenolic compounds are especially produced by plants in response to environmental stresses (Wagay et al., 2020). In the present study, ash application increased moderately the free phenolic acid content, particularly *p*-coumaric acid, in stem and leaves in the hybrid D24 (Figure 4) while there was not a significant impact on the drought-sensitive hybrid P1921. Increased phenolic content observed in D24 due to ash application may not be considered as a stress response as there were no observable negative impacts on plant growth, rather it could be a priming response. It is also noteworthy that a differential response was observed between the hybrids, suggesting that wood ash application would have different effects depending on the variety cultivated and hence, needs to be considered further.

### Elemental Plant Uptake

Uptake of elements was generally disparated between the hybrids and mostly reduced in the stem + leaves compartment in the field experiment. However, increased Ca in cobs, and increased P and Ni in grains of D24 hybrid were observed (Figure 5). Among the basic cations, uptake of Ca and K among others increases noticeably with the application of wood ash, as these elements are present in high concentrations in this amendment (Ohno and Erich, 1990; Erich and Ohno, 1992), as also observed in the ash used in the present study (Table 1). Hence, these highly available elements are generally taken up more readily if not hindered by any competing precipitation and immobilization processes. Supporting our results, except for Mg, a decreasing trend in accumulation was observed for all the analyzed macro and trace elements in the shoot tissue of maize plants grown with biomass fly ash application (Ondrasek et al., 2021). As the availability of minor elements is lower at higher soil pH, hence their plant uptake is often lower. Therefore, high soil pH could be attributed among other confounding factors to the generally low plant uptake of trace elements observed in our study.

### Maize Leaf Transpiration

It was evident from the FTSW calculations that maize hybrid D24 was indeed drought tolerant compared to P1921, as indicated by the stomatal closure/increased stomatal resistance over FTSW (Figure 6). Interestingly, the ash amendment reduced the FTSW threshold at which transpiration started to decline in both hybrids, suggesting that the ash application had a positive effect on leaf transpiration under an artificial progressive water stress regime (Figure 6). Previously, ash application was reported to improve hydraulic conductivity (Chang et al., 1977), increase soil water retention (Pathan et al., 2003; Stoof et al., 2010), and improve water infiltration (Yunusa et al., 2006). These positive effects on soil hydraulic conductivity along with improved root growth observed in our study could be attributed to both the maize hybrids coping well under water stress, as also previously documented in biochar amended soil (Romdhane et al., 2019).

## Conclusion

Results from the present study showed that wood ash is a rich source of nutrients, and maize plants grown in wood ash amended soil had improved the shoot and root growth, the uptake of important elements, and retarded the inhibition of leaf transpiration under drought conditions. Thus, wood ash as soil amendment in our experimental conditions in 1-year trial showed promising results with respect to plant growth and reducing drought sensitivity. Supported by literature, hence, it is demonstrated that wood ash could be one profitable option to counter drought episodes in maize cultivation. Therefore, it will be worthwhile and interesting to assess the impact of wood ash application on growth, yield, and drought tolerance of maize plants cultivated in larger, multi-year field trails with varying drought conditions in the future. Given that ash application in soil can have diverse responses in maize hybrids with differing drought tolerance, focused trials may also be needed to optimize timing, rate, and method of application, specific to the variety cultivated. Beneficial effects of ash application could be maximized when combined with other organic and inorganic amendments as shown earlier in some cases; however, more research is needed to assess its effects in combinations on plant growth and productivity. With no significant adverse effects on plant growth and yield, wood ash can be profitably used as a soil amendment in maize cultivation for improving growth and reducing drought sensitivity. Attention should be payed when using ash derived by metal contaminated wood stocks to avoid any health risk in food uses.

## Data Availability Statement

The data presented in this study are available on request from the corresponding author.

## Author Contributions

TV: conceptualization, methodology, resources, supervision, project administration, and funding acquisition. LRo, CDC, LRa, and LBE: formal analysis. LRo, CDC, and GB: investigation. LRo and LBE: data curation. LRo: writing – original draft preparation. LBE, AP, and TV: writing – review and editing. All authors contributed to the article and approved the submitted version.

### Conflict of Interest

The authors declare that the research was conducted in the absence of any commercial or financial relationships that could be construed as a potential conflict of interest.
