# Supplementary material for: Effects of Soil Amendment With Wood Ash on Transpiration, Growth, and Metal Uptake in Two Contrasting Maize (Zea mays L.) Hybrids to Drought Tolerance
Source: Front Plant Sci. 2021 May 20;12:661909. doi: 10.3389/fpls.2021.661909 (PMC8173060; doi:10.3389/fpls.2021.661909)
Supplement: Supplementary file 1 [file Table_1.DOCX]

Supplementary Material

**Table SI 1.** Field trial, harvest time: Grain yield, protein and lipid contents in kernels of two maize hybrids (D24, P1921) grown under ash-amended soil (Ash, 0.1% w/w) vs. untreated controls (Unt) (mean ± S.E., n=3). Different letters indicate statistically significant differences between treatments within the same hybrid (Newman-Keuls test, P ≤ 0.05).

| **Hybrid** | **Treatment** | **Grain yield**  **(g m^-2^)** | | **Grain proteins**  **(% DW)** | | **Grain lipids**  **(% DW)** | |
| --- | --- | --- | --- | --- | --- | --- | --- |
| **D24** | Unt | 1768±45^a^ |  | 7.57±0.43^a^ |  | 2.76±0.10^a^ |  |
|  | Ash | 1671±31^a^ |  | 7.21±0.18^a^ |  | 2.68±0.02^a^ |  |
| **P1921** | Unt | 1861±22^a^ |  | 6.61±0.06^a^ |  | 2.95±0.04^a^ |  |
|  | Ah | 1858±36^a^ |  | 6.25±0.07^a^ |  | 2.92±0.07^a^ |  |
